# Patient-derived 3D organotypic co-cultures as a long-term ex vivo model for pleomorphic adenoma of the parotid gland

**DOI:** 10.1038/s41598-026-71512-3

**Published:** 2026-09-13

**Authors:** Cosima C. Hoch, Tobias Weiser, Fabian Stögbauer, Felix Johnson, Gabriele Multhoff, Ali Bashiri Dezfouli, Barbara Wollenberg

**Affiliations:** 1 Department of Otolaryngology, Head and Neck Surgery, TUM School of Medicine and Health, Technical University of Munich (TUM), Ismaningerstrasse 22, 81675 Munich, Germany; 2 Institute of Pathology, TUM School of Medicine and Health, Technical University of Munich (TUM), Munich, Germany; 3 Department of Otorhinolaryngology, Head and Neck Surgery, Heidelberg University Hospital, Heidelberg, Germany; 4 Central Institute for Translational Cancer Research, Technical University of Munich (TranslaTUM), Munich, Germany

**Keywords:** Pleomorphic adenoma, Parotid gland, Organotypic co-culture, Ex vivo model, Three-dimensional (3D) culture, Immunohistochemistry, Cancer, Oncology

## Abstract

Pleomorphic adenoma is the most common benign salivary gland tumor and most frequently arises in the parotid gland. Although benign, it can recur and may undergo malignant transformation, underscoring the need for experimental models that preserve native tissue features over time. In this study, fresh tumor tissue from six patients with primary histologically benign pleomorphic adenoma was divided into fragments of approximately 3 × 3 mm in surface dimensions and approximately 3 mm in thickness and maintained in a patient-derived three-dimensional organotypic co-culture for up to 21 days. The primary longitudinal analysis compared baseline tissue with matched fragments harvested at days 14 and 21. Histology and immunohistochemistry were assessed in three randomly selected high-power fields within morphologically viable, tumor-containing regions; necrotic or degenerative areas were excluded. Ki-67-positive cell nuclei remained within the predefined low category at all retained time points. PLAG1 positivity was retained in all cases but was more variable by day 21, while epithelial and myoepithelial-associated markers showed marker- and case-dependent patterns. CD45-positive cells remained detectable in sampled viable compartments, whereas SSTR2 was commonly reduced after baseline. These findings support the use of the 3D-OTC platform for longitudinal histomorphologic and selected biomarker assessment within surviving viable tissue compartments. Because whole-fragment necrosis and diffusion of oxygen, nutrients, or test compounds were not quantified, the present data do not establish uniform full-thickness viability or validate the platform for therapeutic-response testing.

**Supplementary Information:**

The online version contains supplementary material available at https://doi.org/10.1038/s41598-026-71512-3.

## Introduction

Pleomorphic adenoma (PA) is the most common benign salivary gland tumor and arises most frequently in the parotid gland^1^. Although PA is classified as benign, it is clinically relevant because it can recur, may show multifocal growth patterns, and can undergo malignant transformation to carcinoma ex pleomorphic adenoma in a subset of cases^2^. Standard management is surgical resection, but treatment decisions and follow-up strategies benefit from a better understanding of tumor biology and from translational tools that allow testing of diagnostic and therapeutic concepts under controlled conditions^3^.

PA displays pronounced histologic heterogeneity. It is typically composed of epithelial and myoepithelial-like tumor components arranged in duct-like structures, nests, and trabeculae, embedded in variably myxoid to chondromyxoid stroma^4^. This complex organization is central to PA pathology, but it also complicates experimental modeling. Conventional 2D cell culture systems often fail to preserve tissue architecture, stromal context, and differentiation states^5,6^. While organoid/tumoroid approaches and slice cultures have expanded the toolbox for salivary gland research, accessible and robust patient-derived ex vivo platforms for PA that enable longitudinal readouts remain limited^7,8^. This gap is particularly relevant when the research goal is translational evaluation of biomarker stability or receptor targets in a tissue context.

One promising translational direction in PA is somatostatin receptor 2 (SSTR2). Our group previously demonstrated that SSTR2 can be expressed in PA and may be associated with clinically relevant imaging using somatostatin-analog–based PET/CT, supporting the concept that SSTR2 could serve as a diagnostic target and, potentially, as a basis for future targeted approaches^9–11^. However, translation requires model systems that preserve tumor identity and differentiation features over time and that allow systematic evaluation of marker stability under ex vivo conditions^12–14^. Without such models, it remains difficult to determine whether target expression is robust enough for experimental manipulation and how culture conditions might affect receptor phenotypes.

Here, we establish a patient-derived three-dimensional organotypic co-culture (3D-OTC) model for PA of the parotid gland using fibroblast-based dermal equivalents (DEs) as a standardized supportive scaffold rather than as a salivary-gland-specific reconstruction of the tumor microenvironment. We aimed to determine whether PA-defining histomorphology and selected immunophenotypic features can be assessed longitudinally in morphologically viable regions during ex vivo culture. The primary longitudinal analysis compared baseline tissue with matched fragments harvested at days 14 and 21 for proliferation (Ki-67), tumor identity (PLAG1), epithelial differentiation and cohesion (PanCK and E-cadherin), myoepithelial/basal-associated features (α-SMA and p63), resident leukocytes (CD45), and the exploratory translational target SSTR2.

## Materials and methods

### Patient cohort

This study included six patients with pleomorphic adenoma of the parotid gland (*n* = 6; three female and three male) undergoing primary surgery at the Department of Otorhinolaryngology, Head and Neck Surgery, TUM Klinikum, Technical University of Munich. The mean age at diagnosis was 62.2 ± 15.7 years. Eligibility required sufficient resection material for organotypic co-culture and histopathologic assessment. Histopathologic diagnoses were established using the routine diagnostic material. Tissue collection and clinical-data acquisition were approved by the Ethics Committee of the School of Medicine at the Technical University of Munich (2022 − 591_1-S-KH) and performed in accordance with the Declaration of Helsinki. All patients provided written informed consent, and patient data were anonymized prior to analysis. Demographic characteristics are summarized in Table S1.

### Fibroblast culture and generation of dermal equivalents

Primary normal human adult dermal fibroblasts (HDFa; PCS-201-012™, ATCC) were used to generate dermal equivalents (DEs). Fibroblasts were cultured on sterile 11-mm discs of non-woven viscose fabric (#Jettex 2005/45, 45 g/m² spunlace, 100% viscose; Orsa NW s.r.l., Italy) placed in 12-well filter inserts (polyester membrane; 2-µm pore size; #665641, Greiner Bio One, Austria). For each construct, fibroblasts were embedded in a thrombin (#T6884-250UN, Merck, Germany)/fibrinogen (#F3879-250MG, Merck, Germany) mixture and polymerized at 37 °C. Constructs were maintained in DMEM (#21885108, ThermoFisher Scientific, USA) supplemented with 10% fetal bovine serum (#A5256801, ThermoFisher Scientific, USA), 1% penicillin/streptomycin (#15140122, ThermoFisher Scientific, USA), TGF-β (1 ng/mL; #7754-BM-005, R&D Systems, USA), and L-ascorbic acid (50 µg/mL; #6288.1, Carl ROTH, Germany), with medium changes every 3–4 days. DEs were pre-cultured for 7–14 days to permit extracellular-matrix deposition and scaffold stabilization. This DE platform has previously been used as a standardized support for organotypic head-and-neck tumor models^15^; in the present study it served as a supportive scaffold and was not intended to reproduce salivary-gland-specific stroma.

### Tissue processing and organotypic co-culture

**Fig. 1:**
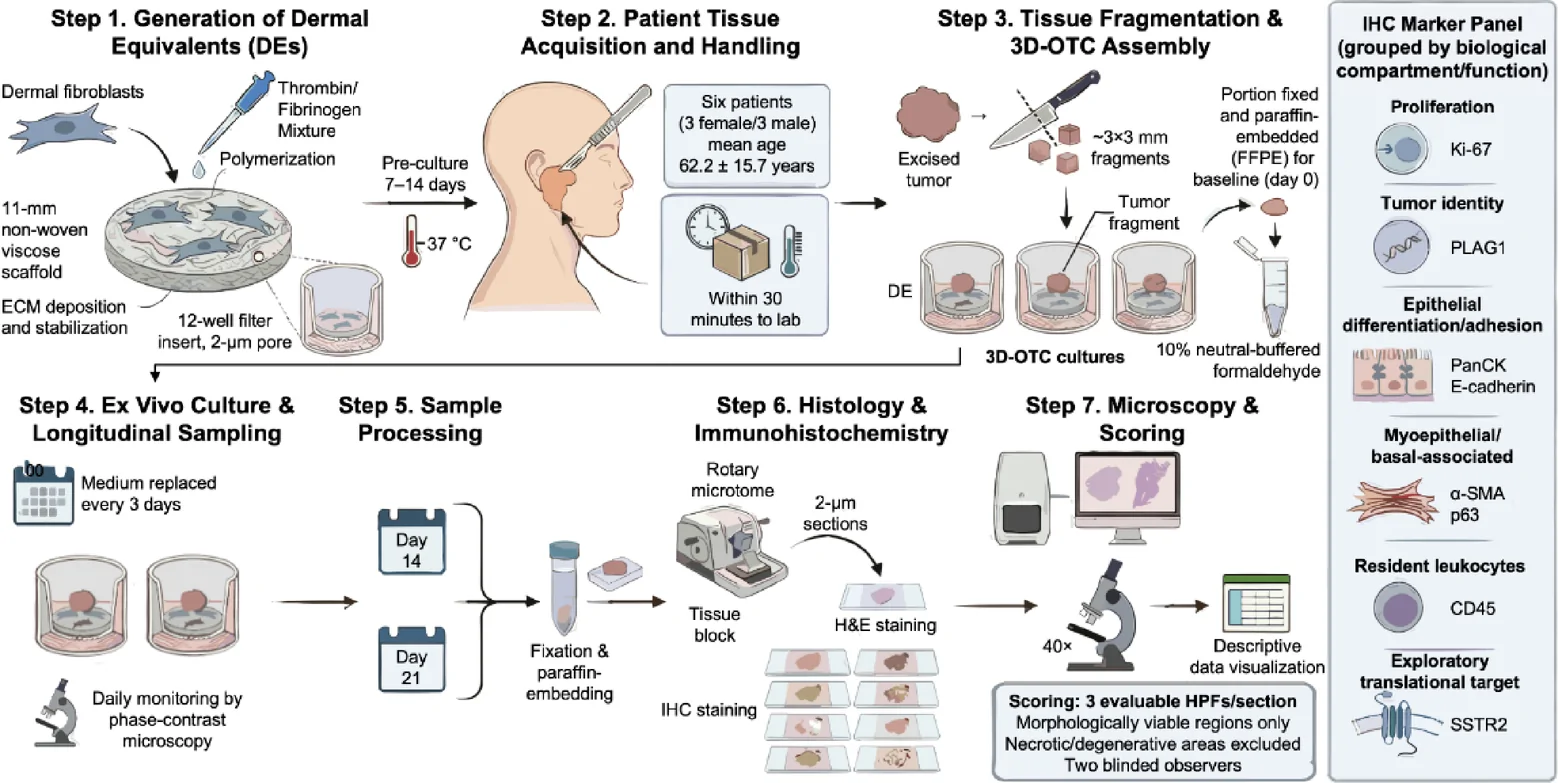
Workflow schematic of the patient-derived 3D organotypic co-culture (3D-OTC) model for pleomorphic adenoma (PA) of the parotid gland and downstream analyses. Step 1: Dermal equivalents (DEs) were generated using primary normal human adult dermal fibroblasts on an 11-mm non-woven viscose scaffold and pre-cultured for 7–14 days. Step 2: Fresh PA tissue was obtained during surgery (*n* = 6), transported to the laboratory within 30 min, and processed without cryopreservation or extended storage. Step 3: Tumor tissue was manually divided into fragments of approximately 3 × 3 mm in surface dimensions and approximately 3 mm in thickness and placed on the DEs; a separate portion was immediately fixed as the primary-tumor baseline control (day 0). Steps 4–5: 3D-OTCs were maintained in supplemented DMEM/F12, with medium replacement every 3 days, and separate matched fragments were harvested at predefined culture time points. The primary longitudinal analysis compared baseline, day 14, and day 21. Steps 6–7: Formalin-fixed, paraffin-embedded sections underwent H&E staining and immunohistochemistry for Ki-67, PLAG1, PanCK, E-cadherin, α-SMA, p63, CD45, and SSTR2. For each marker and retained time point, three randomly selected, non-overlapping high-power fields within morphologically viable, tumor-containing regions were evaluated independently by two blinded observers; necrotic or degenerative areas were excluded. Marker-specific scoring and field-level aggregation are described in the Methods. This workflow schematic was created using a Canvas-based illustration template.

Following definitive surgical resection, fresh PA tissue was collected immediately and transported to the laboratory within 30 min of excision. No cryopreservation or extended storage was performed before culture. Tumor tissue was manually sectioned with a scalpel into fragments of approximately 3 × 3 mm in surface dimensions and approximately 3 mm in thickness and placed on the pre-established DEs. The exact central-versus-peripheral intratumoral origin of individual fragments was not documented. Samples were cultured in 5 mL of DMEM/F12 (1:1; #31765035, ThermoFisher Scientific, USA) supplemented with 10% fetal bovine serum, 1% penicillin/streptomycin, aprotinin (8.3 µg/mL; #A162.2, Carl ROTH, Germany), and L-ascorbic acid (50 µg/mL; #6288.1, Carl ROTH, Germany). Cultures were monitored daily by phase-contrast microscopy and medium was replaced every 3 days. On the day of resection, a portion of PA tissue was immediately fixed in 10% neutral-buffered formaldehyde for 1 h at room temperature, transferred to 70% ethanol, processed (Leica ASP300S, Leica Biosystems, Wetzlar, Germany), and paraffin-embedded as the baseline control (day 0). Separate physical fragments derived from the same patient were harvested at the predefined culture time points, fixed, and paraffin-embedded; thus, successive time points represent matched fragments rather than repeated measurements of the same physical specimen. All available patient-derived fragments were used for the predefined baseline or culture analyses, and no unfixed material remained for retrospective molecular assays. The workflow is summarized in Fig. 1.

### Histology and immunohistochemistry

For histologic evaluation of tissue morphology and integrity, formalin-fixed paraffin-embedded (FFPE) tissue blocks from all time points were sectioned at 2 μm using a rotary microtome (HM355S, ThermoFisher Scientific, Waltham, USA) and mounted on Superfrost glass slides. Sections were deparaffinized in xylene, rehydrated through graded alcohols, and stained with hematoxylin and eosin (H&E) according to standard protocols. Slides were then dehydrated through graded alcohols, cleared in xylene, and coverslipped using Pertex mounting medium (Histolab, Gothenburg, Sweden).

Immunohistochemistry (IHC) was performed on FFPE sections using automated staining platforms (Bond RXm; Leica Biosystems, Wetzlar, Germany; and BenchMark XT; Ventana Medical Systems, Tucson, AZ, USA) following the manufacturers’ protocols. Sections were deparaffinized (Leica De-Wax kit or Ventana EZ Prep), subjected to heat-induced epitope retrieval, and endogenous peroxidase activity was blocked (Bond RXm: 3% hydrogen peroxide; BenchMark XT: UV inhibitor). Slides were incubated with the respective primary antibodies (Bond RXm: 15 min at room temperature; BenchMark XT: 20 min at 37 °C), followed by polymer-based detection (EnVision HRP rabbit polymer; Dako, Glostrup, Denmark) or, where applicable, biotinylated secondary antibodies with streptavidin-peroxidase (Medac Diagnostica, Wedel, Germany). Signal was visualized using 3,3′-diaminobenzidine (DAB; Medac Diagnostica, Wedel, Germany), and sections were counterstained with hematoxylin (BenchMark XT including a subsequent bluing step). Slides were dehydrated, cleared in xylene, and coverslipped using Pertex mounting medium (Histolab, Gothenburg, Sweden).

A marker panel was applied to characterize native PA tissue and corresponding 3D-OTCs, including Ki-67 (proliferation), PLAG1 (tumor-associated marker), PanCK and E-cadherin (epithelial differentiation and adhesion), α-SMA and p63 (myoepithelial/mesenchymal and basal-associated populations), CD45 (resident leukocyte assessment), and SSTR2 (exploratory translational target marker). A complete list of primary antibodies and staining conditions is provided in Table S2.

### Microscopy and semi-quantitative scoring

To account for intratumoral heterogeneity, three randomly selected, non-overlapping high-power fields per section were assessed at 40× magnification within morphologically viable, tumor-containing regions. Necrotic or degenerative areas were excluded from all scoring, and staining hotspots were not preferentially targeted. Microscopic evaluation was performed using a Leica Aperio AT2 scanner and Aperio ImageScope software. Scoring was conducted independently by two blinded observers (C.C.H. and F.S.), and discrepancies were resolved by consensus. The original analysis did not include whole-section morphometric quantification of the non-viable fraction.

Proliferative activity was recorded as the percentage of Ki-67-positive cell nuclei among all nuclei within the evaluated viable region and categorized as low (≤ 15%), intermediate (15–≤30%), or high (> 30%). Because no lineage co-staining was performed, Ki-67-positive nuclei were not uniformly attributed to tumor cells.

PLAG1 expression was scored according to the percentage of PLAG1-positive tumor cell nuclei as − (≤10%), + (>10–≤50%), ++ (>50–≤80%), or +++ (>80%)^16^.

PanCK and α-SMA were evaluated based on cytoplasmic staining of tumor cells and categorized as − (no staining), + (< 10% positive tumor cells), ++ (10–50%), or +++ (> 50%). E-cadherin was scored based on membranous staining of tumor cells as − (no staining or ≤ 10% positivity), + (incomplete weak membranous staining in > 10%), ++ (complete weak-to-moderate membranous staining in > 10%), or +++ (complete strong membranous staining in > 10%)^17^. p63 expression was evaluated using a combined score incorporating staining intensity and the proportion of positive nuclei: − (no staining), + (1 + in ≤ 70% or 2 + in ≤ 30%), ++ (1 + in > 70%, 2 + in >30% and ≤70%, or 3 + in ≤ 30%), and +++ (2+ in >70% or 3+ in >30% of cells)^18^.

CD45 was assessed as the percentage of CD45-positive cells among all cells within the tissue and categorized as + (>0% and <10%), ++ (≥10% and <40%). Only cells with a clearly discernible membranous and/or cytoplasmic signal and a vital nucleus were considered positive.

SSTR2 expression was evaluated using a modified HER2-like scoring system based on intensity and distribution of membranous staining: − (no/weak staining in ≤ 10%), + (incomplete weak staining in > 10%), ++ (incomplete or strong circumferential staining in > 10% or strong staining in ≤ 10%), and +++ (complete strong staining in > 10%)^19^.

### Statistical analysis

Given the exploratory and descriptive nature of this study and the small cohort (*n* = 6), no formal hypothesis testing was performed. For each specimen, marker, and time point, the three high-power-field measurements were averaged. During final quality control of the expanded longitudinal dataset, day-7 observations showed internally discordant cross-marker patterns that could not be biologically interpreted with confidence. The primary longitudinal analysis was therefore restricted to baseline (day 0), day 14, and day 21; day-7 values were excluded from the primary interpretation and from the supplementary quantitative tables. Continuous percentage data are summarized as mean ± standard deviation and median (range) across the six independent cases. Ordinal categorical scores (−/+/++/+++) are presented primarily as medians, ranges, and category distributions. For markers with an applicable ordinal score, scores were also numerically encoded (0–3) to provide secondary descriptive mean ± standard deviation values in Table S4; these means were not treated as continuous inferential outcomes. Ki-67 was summarized using its predefined low/intermediate/high categories rather than the −/+/++/+++ system. Individual specimen-level values are provided in Table S3.

## Results

### Histomorphology and proliferative index

The primary objective of this analysis was to determine whether PA-specific morphological hallmarks are preserved in the 3D-OTC model throughout the culture period. Histologically, PA is characterized by a biphasic epithelial-myoepithelial tumor component arranged in duct-like epithelial structures, nests, and trabeculae, embedded in a variable myxoid to chondromyxoid stroma, resulting in marked architectural heterogeneity. These features served as reference criteria to evaluate morphological preservation in culture.

**Fig. 2:**
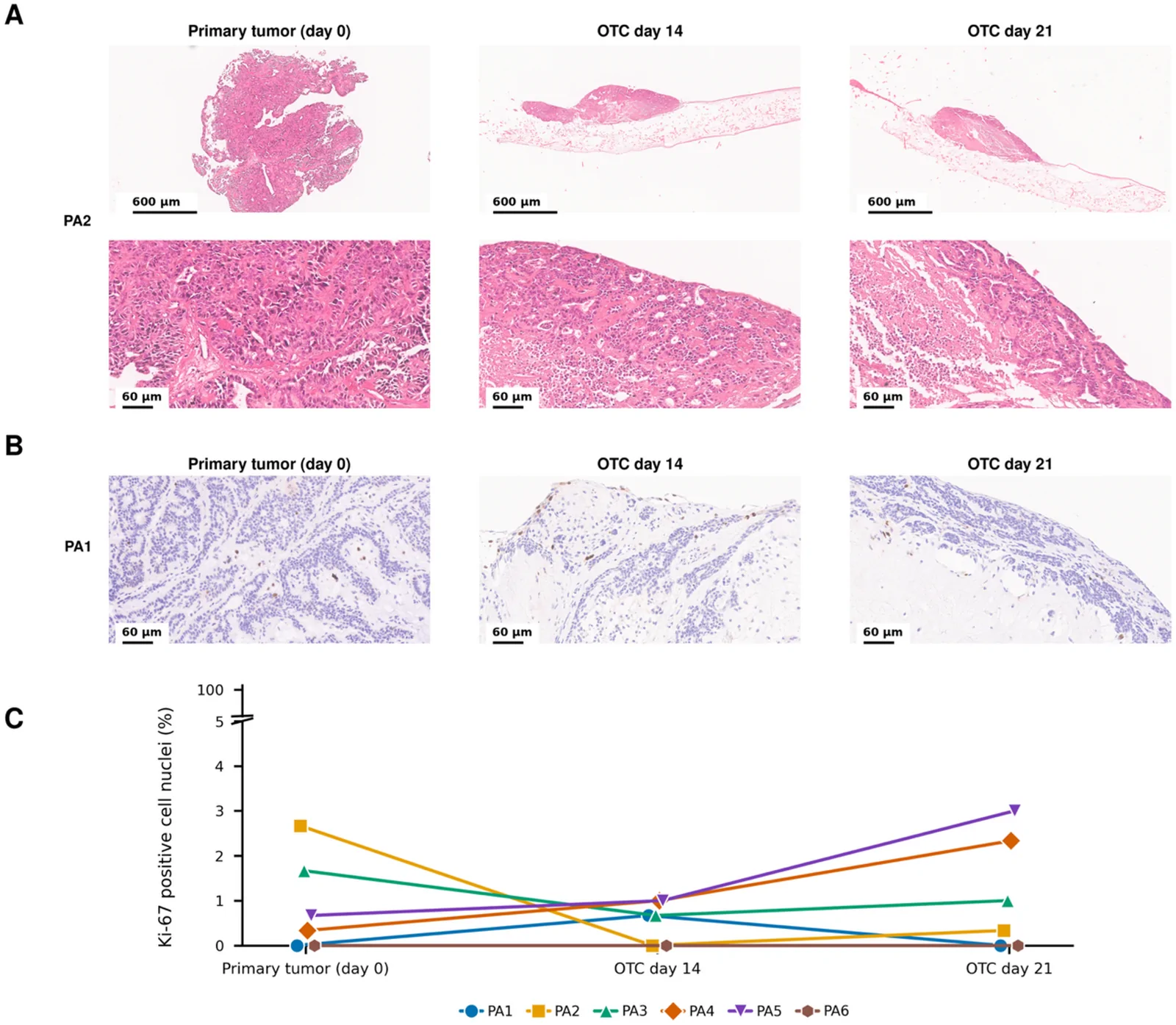
Preservation of histomorphology and proliferative activity during 3D organotypic co-culture. (**A**) Representative H&E-stained sections from PA2 at baseline (primary tumor, day 0) and OTC days 14 and 21. The 4× overviews show the tissue fragments and their relationship to the supporting scaffold; the corresponding 40× images show morphologically viable tumor-containing regions. (**B**) Representative Ki-67 immunohistochemistry in PA1 at the retained time points; brown nuclear staining denotes Ki-67-positive nuclei, with hematoxylin counterstaining. (**C**) Individual percentages of Ki-67-positive cell nuclei in six independent PAs. Connected symbols link matched fragments derived from the same biological case. Values are specimen-level means from three randomly selected high-power fields within morphologically viable regions. Scale bars, 600 μm (4×) and 60 μm (40×).

H&E staining was examined in all six primary tumors and corresponding 3D-OTCs at days 14 and 21. Within the morphologically viable areas selected for evaluation, characteristic epithelial–myoepithelial architecture and stromal features remained recognizable at the retained culture time points. The representative PA2 series additionally demonstrates the relationship between the cultured tumor fragment and the supporting scaffold at low magnification and the preserved morphology of selected viable areas at high magnification (Fig. 2A). Some 3D-OTCs contained focal central degenerative or necrotic-appearing regions, which were excluded from IHC scoring. Because a standardized whole-section estimate of the non-viable fraction was not acquired, these observations document recognizable morphology in sampled viable compartments but do not establish uniform viability throughout the complete fragment.

Ki-67 IHC was quantified in all six cases at baseline and days 14 and 21 (Fig. 2B,C). Mean Ki-67-positive cell nuclei were 0.89 ± 1.07% at baseline, 0.56 ± 0.46% at day 14, and 1.11 ± 1.28% at day 21. Although individual matched fragments varied, every specimen remained within the predefined low category (≤ 15%) at all retained time points (Tables S3 and S4). These data describe persistence of a low proliferative fraction in the evaluated viable regions; Ki-67 alone is not a direct measure of cell viability and does not demonstrate survival across the complete fragment.

### PLAG1 immunoreactivity

PLAG1 is a tumor-associated marker that is typically overexpressed in PA and represents a central feature in the molecular characterization of these tumors. To evaluate tumor-associated properties within the 3D-OTC model, PLAG1 expression was assessed by IHC in the primary tumors and corresponding matched fragments harvested after 14 and 21 days^16^.

**Fig. 3:**
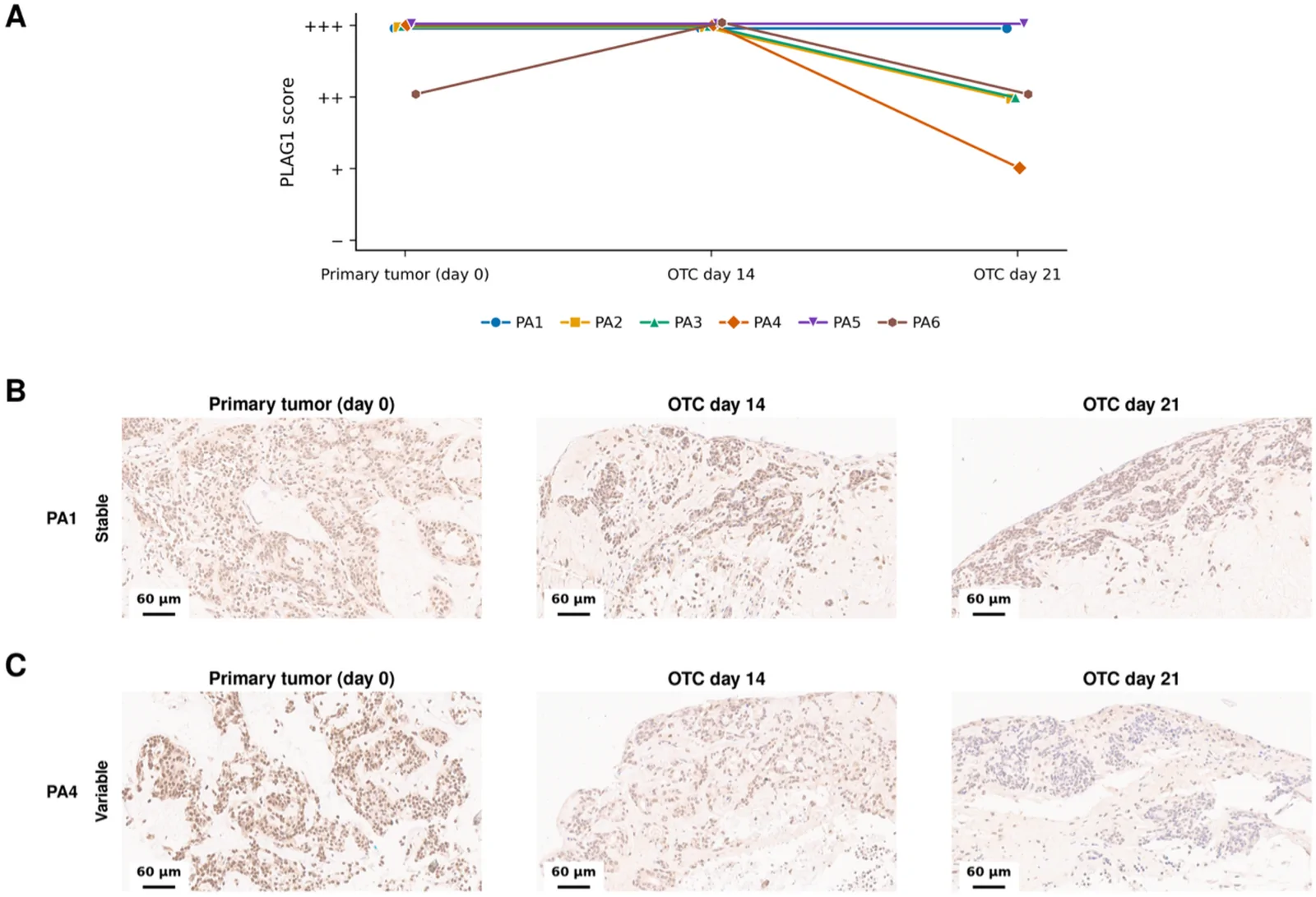
PLAG1 expression during 3D organotypic co-culture. (**A**) Individual PLAG1 scores for the six independent PAs at baseline (primary tumor, day 0) and OTC days 14 and 21. Connected symbols link matched fragments derived from the same biological case. (**B**,** C**) Representative PLAG1 immunohistochemistry at the retained time points in PA1 (stable) and PA4 (variable, with a lower score at day 21). Brown nuclear staining indicates PLAG1 immunoreactivity; sections were counterstained with hematoxylin. Ordinal scores were assigned according to the marker-specific criteria described in the Methods. Scale bars, 60 μm.

PLAG1 remained detectable in all six cases at baseline and days 14 and 21, but patterns differed among matched fragments (Fig. 3A). Mean PLAG1-positive nuclei were 91.00 ± 8.40% at baseline, 92.42 ± 3.62% at day 14, and 75.94 ± 21.14% at day 21. At day 21, scores were +++ in two cases, ++ in three cases, and + in one case. PA1 illustrates consistently high expression across the retained fragments, whereas PA4 illustrates a variable pattern with lower expression in the day-21 fragment (Fig. 3B,C; Tables S3 and S4). Thus, PLAG1 positivity was retained in all evaluated cases, but uniformly high expression was not maintained in every fragment.

### Epithelial differentiation and cell-cell adhesion

To assess epithelial differentiation and cell-cell cohesion during long-term ex vivo culture, we evaluated PanCK and E-cadherin expression by IHC in primary tumors (day 0) and corresponding matched fragments harvested at days 14 and 21. PanCK was selected as a broad epithelial marker to capture the epithelial tumor component, whereas E-cadherin was assessed as a key mediator of epithelial cell-cell adhesion and tissue cohesion^20–22^.

**Fig. 4:**
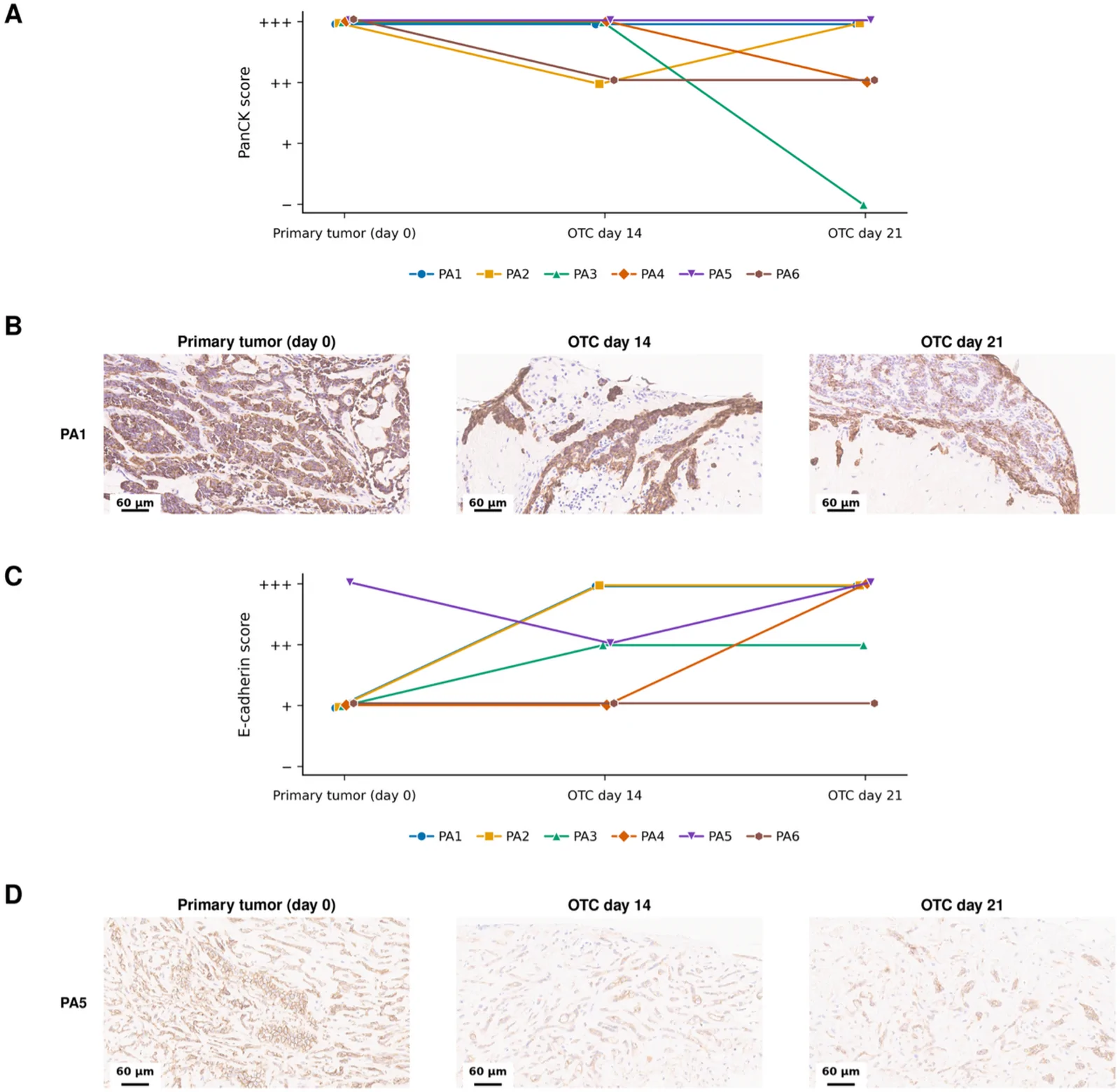
Epithelial marker expression during 3D organotypic co-culture. (**A**) Individual PanCK scores for the six PAs at baseline (primary tumor, day 0) and OTC days 14 and 21. (**B**) Representative PanCK immunohistochemistry in PA1 at the retained time points. (**C**) Individual E-cadherin scores for the six PAs across the same time points. (**D**) Representative E-cadherin immunohistochemistry in PA5. Brown cytoplasmic staining denotes PanCK immunoreactivity, whereas brown membranous staining denotes E-cadherin immunoreactivity; sections were counterstained with hematoxylin. Connected symbols link matched fragments derived from the same biological case. Ordinal scores were assigned according to the marker-specific criteria described in the Methods. Complete additional series illustrating inter-case variability are shown in Supplementary Fig. S1. Scale bars, 60 μm.

All six primary tumors had a PanCK score of +++ at baseline. PanCK remained detectable in all six matched fragments at day 14 and in five of six at day 21, but the fragment-level patterns varied substantially (Fig. 4A). At day 21, three cases were +++, two were ++, and one was negative. PA1 is shown as a comparatively stable representative series, whereas the variable PA3 series is provided in Supplementary Fig. S1 (Fig. 4B; Tables S3 and S4). The retained longitudinal data therefore support broad persistence of PanCK positivity in most, but not all, viable fragments rather than uniform marker stability.

E-cadherin membranous immunoreactivity was detectable in all six primary tumors and in all six matched fragments at days 14 and 21 (Fig. 4C). The score distribution shifted toward higher categories at the later retained time points, but individual patterns remained case-dependent and must be interpreted in the context of fragment-to-fragment heterogeneity. PA5 is shown in the main figure, and the variable PA6 series is shown in Supplementary Fig. S1 (Fig. 4D; Tables S3 and S4).

### Myoepithelial and mesenchymal cell populations

To characterize myoepithelial and mesenchymal-associated compartments within the 3D-OTC model, α-SMA and p63 expression were assessed by IHC in primary tumors (day 0) and corresponding matched fragments harvested after 14 and 21 days. α-SMA was used to visualize myoepithelial/stromal components, whereas p63 was evaluated as a nuclear marker associated with basal/myoepithelial differentiation in PA^23,24^.

**Fig. 5:**
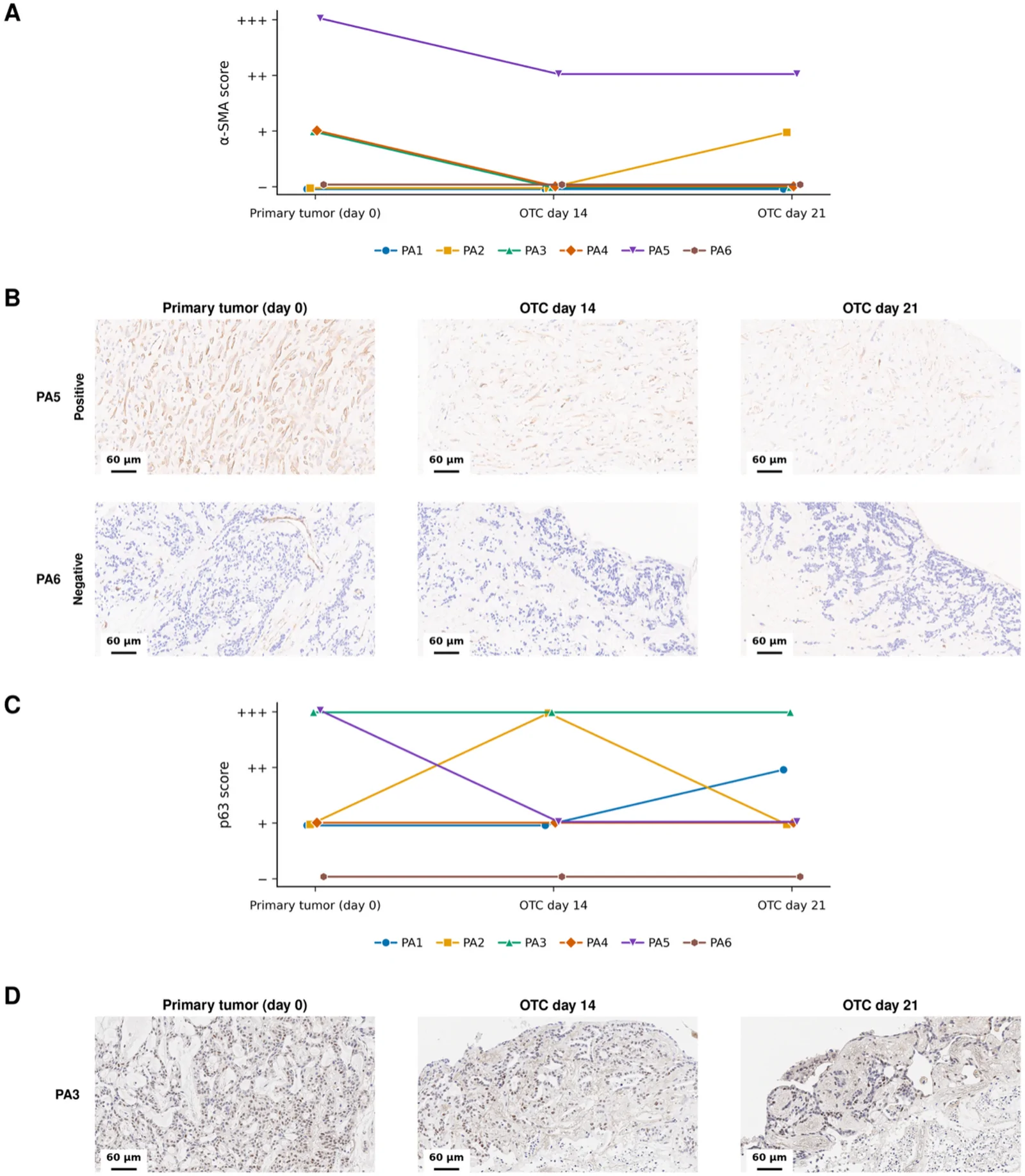
Myoepithelial-associated marker expression during 3D organotypic co-culture. (**A**) Individual α-SMA scores for the six PAs at baseline (primary tumor, day 0) and OTC days 14 and 21. (**B**) Representative α-SMA immunohistochemistry in PA5 (positive) and PA6 (negative), reflecting inter-case heterogeneity and the predominantly negative/low α-SMA pattern in the cohort. An α-SMA-positive vascular profile is visible in the PA6 primary tumor and serves as an internal positive staining control. (**C**) Individual p63 scores for the six PAs across the retained time points. (**D**) Representative p63 immunohistochemistry in PA3. Brown cytoplasmic staining denotes α-SMA immunoreactivity, whereas brown nuclear staining denotes p63 immunoreactivity; sections were counterstained with hematoxylin. Connected symbols link matched fragments derived from the same biological case. Ordinal scores were assigned according to the marker-specific criteria described in the Methods. Scale bars, 60 μm.

α-SMA staining was predominantly absent or low and showed marked inter-case variability (Fig. 5A). At baseline, three cases were negative, two were +, and one (PA5) was +++; at day 21, four cases were negative, one was +, and one was ++. The main figure therefore shows PA5 as a positive example and PA6 as a negative example (Fig. 5B), thereby representing both the less frequent positive pattern and the predominantly negative/low cohort pattern. In the α-SMA-negative PA6 primary tumor, an α-SMA-positive vascular profile serves as an internal positive staining control and supports the technical validity of the negative tumor-cell readout (Tables S3 and S4).

p63 was detectable in five of six cases at baseline and at both retained culture time points, whereas PA6 remained negative (Fig. 5C). Scores differed among the positive matched fragments, but detection in five cases at each retained time point indicates more consistent p63 positivity than α-SMA positivity within the evaluated viable regions. PA3 provides the strong positive representative series in the main figure, while PA6 illustrates the negative pattern in Supplementary Fig. S1 (Fig. 5D; Tables S3 and S4).

### Resident CD45-positive cells

To assess resident leukocytes in the sampled PA fragments, CD45 IHC was performed on primary tumors and corresponding matched fragments at days 14 and 21^25^. Because the cultures were non-vascularized, this readout was not interpreted as immune-cell recruitment, immune expansion, or preservation of a functional immune microenvironment.

**Fig. 6:**
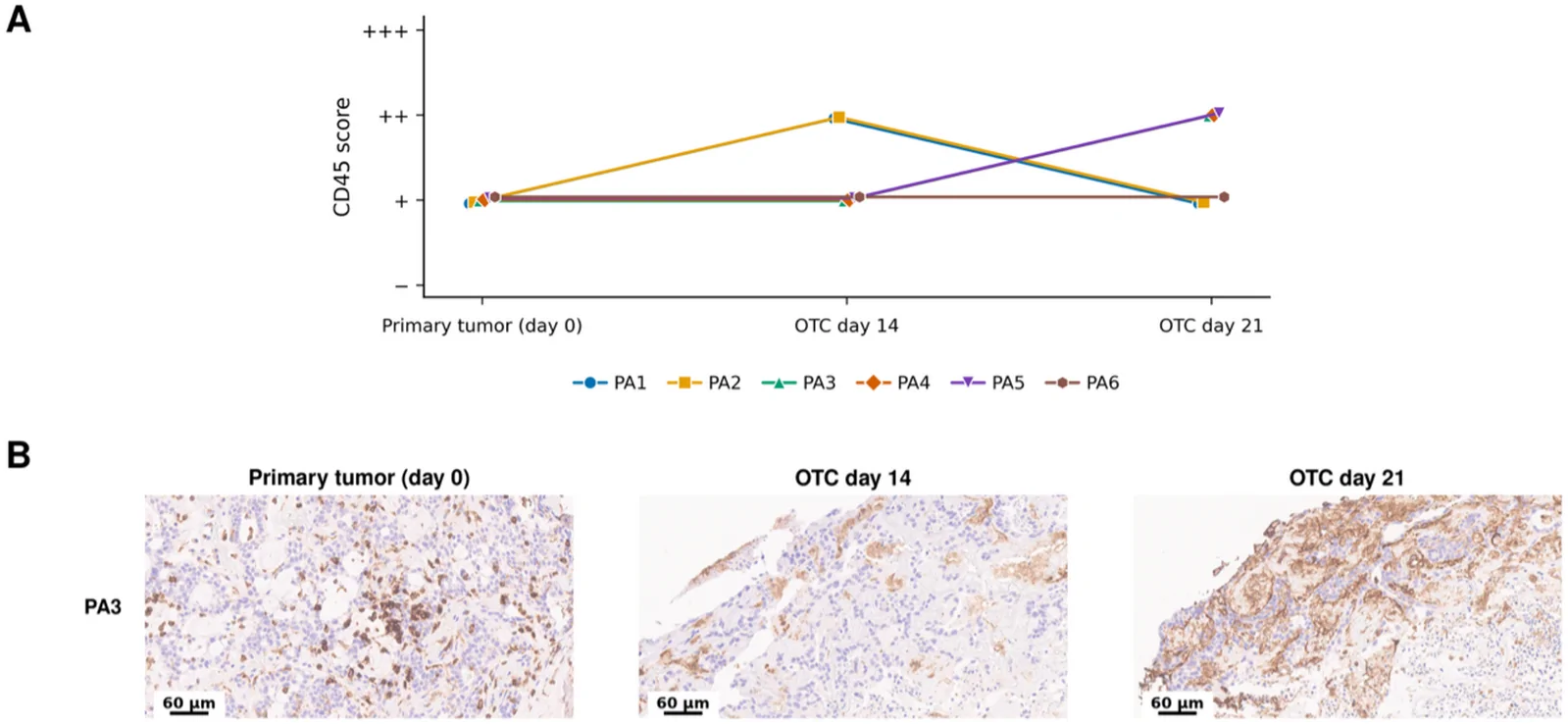
Resident CD45-positive cells during 3D organotypic co-culture. (**A**) Individual CD45 scores for the six PAs at baseline (primary tumor, day 0) and OTC days 14 and 21. Connected symbols link matched fragments derived from the same biological case. (**B**) Representative CD45 immunohistochemistry in PA3 at the retained time points, illustrating that CD45-positive cells remained detectable within sampled viable tumor-containing compartments. Brown membranous and/or cytoplasmic staining identifies CD45-positive cells; sections were counterstained with hematoxylin. Scores were assigned according to the criteria described in the Methods. Scale bars, 60 μm.

CD45-positive cells were detected in all six baseline fragments and remained detectable in all six matched fragments at days 14 and 21 (Fig. 6A). Mean percentages were 5.06 ± 2.63% at baseline, 6.22 ± 7.35% at day 14, and 14.72 ± 11.10% at day 21. A higher percentage of CD45-positive cells does not necessarily indicate an increase in the absolute number of immune cells. In this non-vascularized system, differences may instead reflect fragment composition, sampling location, or relative loss of other cell populations, thereby changing the denominator. PA3 provides a representative series of matched fragments (Fig. 6B; Tables S3 and S4). Because the exact central-versus-peripheral origin of the fragments was not documented, a potential tumor-edge contribution can neither be confirmed nor excluded.

### Exploratory assessment of SSTR2 as a translational marker in PAs

Based on prior work from our group demonstrating SSTR2 expression in PAs, we investigated whether this translationally relevant receptor target for somatostatin-analog-based imaging/therapy remained detectable in matched fragments during long-term ex vivo culture. SSTR2 IHC was performed on primary tumor tissue (day 0) and corresponding fragments harvested at days 14 and 21.

**Fig. 7:**
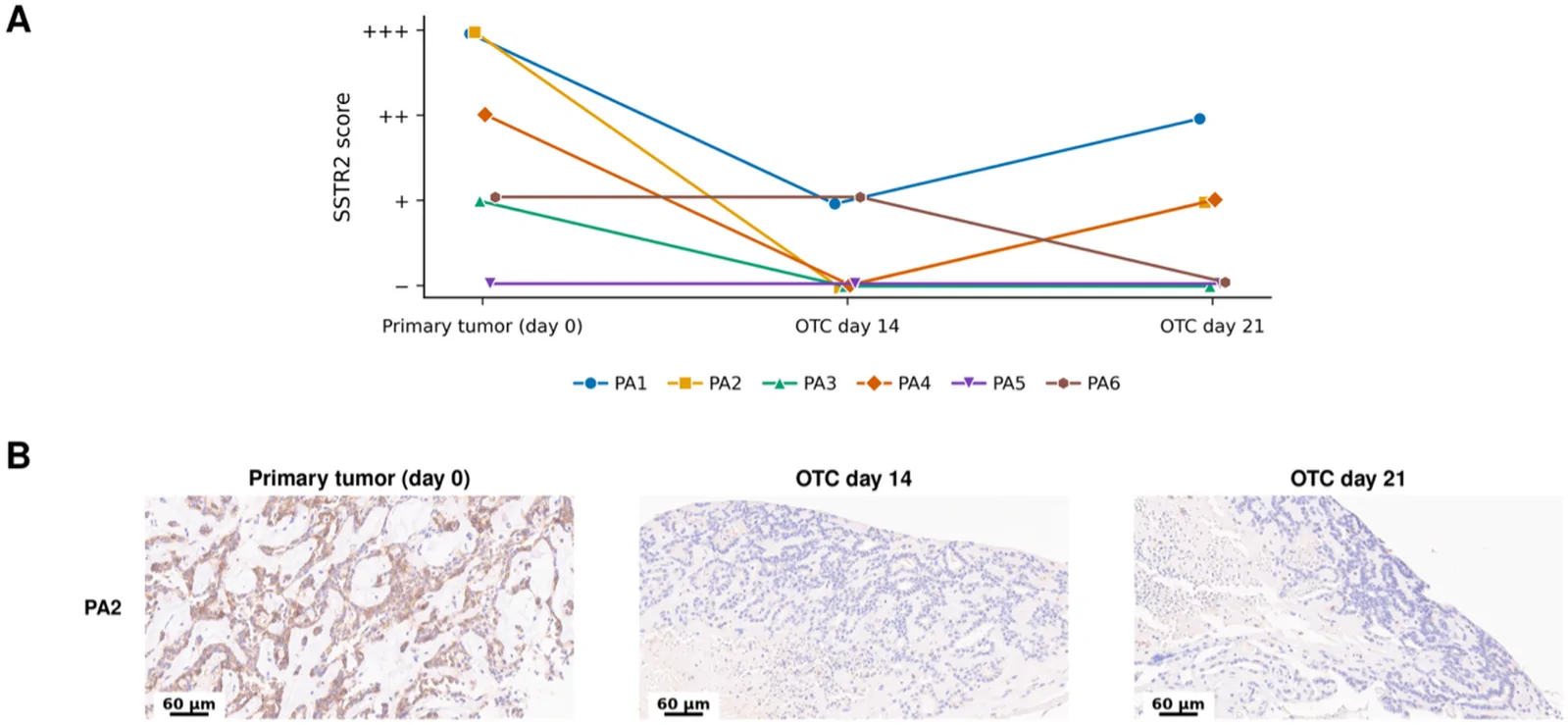
SSTR2 expression during 3D organotypic co-culture. (**A**) Individual SSTR2 scores for the six PAs at baseline (primary tumor, day 0) and OTC days 14 and 21. Connected symbols link matched fragments derived from the same biological case. (**B**) Representative SSTR2 immunohistochemistry in PA2 at the retained time points, showing strong baseline membranous staining and reduced staining in cultured fragments. Brown membranous staining denotes SSTR2 immunoreactivity; sections were counterstained with hematoxylin. Ordinal scores were assigned according to the marker-specific criteria described in the Methods. Scale bars, 60 μm.

SSTR2 positivity differed between matched fragments at the retained culture time points and was commonly attenuated after baseline (Fig. 7A). Five of six primary tumors were positive, compared with two of six fragments at day 14 and three of six fragments at day 21; later positive scores were generally lower than at baseline. PA2 illustrates strong baseline membranous staining and markedly lower staining in the corresponding cultured fragments (Fig. 7B; Tables S3 and S4). Overall, SSTR2 was not reliably maintained under the present culture conditions.

## Discussion

Patient-derived experimental models for PA remain limited, despite the clinical need for systems that preserve tumor architecture and enable analyses beyond static histology. Ex vivo organotypic approaches are increasingly used across tumor entities because they can retain tissue architecture and some stromal and cellular elements carried with the specimen, although the degree of microenvironmental preservation depends on the platform and culture conditions^12,14,15^. Within salivary-gland research, workflows for patient-derived organotypic tissue slices and complementary 3D platforms (e.g., organoids/tumoroids) have shown that salivary tissues can be maintained ex vivo while retaining selected histologic and molecular features^7,26,27^. Building on this rationale, we developed a PA-specific 3D-OTC platform supported by fibroblast-based DEs and maintained patient-derived fragments for up to 21 days. The DE provided standardized structural support; it should not be regarded as a reconstruction of salivary-gland-specific stroma or of the complete native PA microenvironment. Within the morphologically viable compartments evaluated at baseline and the retained culture time points, the platform enabled longitudinal assessment of histomorphology and selected marker expression. A major translational motivation was SSTR2, which our group previously linked to PA biology and clinically relevant imaging (e.g., 68Ga-DOTATOC PET/CT uptake)^9,10^. Overall, the data indicate preservation of recognizable PA morphology, a low proliferative fraction, and PLAG1 positivity, whereas epithelial, myoepithelial-associated, CD45, and receptor-associated readouts differed by marker, case, and sampled fragment.

From a translational perspective, longitudinal patient-derived 3D organotypic models can complement the single time-point snapshot provided by routine histopathology. In the present design, separate patient-matched fragments were allocated to baseline and predefined culture time points; the same physical fragment was therefore not followed repeatedly. Nevertheless, comparison across matched fragments permits assessment of whether diagnostic or exploratory biomarkers remain detectable under defined culture conditions and makes culture-associated variability visible. The present data support this descriptive use for histomorphologic and biomarker-stability studies in surviving viable compartments. They do not yet validate the model for drug-response testing, because spatial penetration of oxygen, nutrients, or test compounds and viability across the full fragment thickness were not measured. Direct tracer-penetration and spatial viability studies, ideally combined with a more salivary-specific stromal context, will be required before the platform can serve as an intermediate therapeutic-testing system. This distinction is particularly important for phenotype-sensitive targets such as SSTR2, whose loss in culture may reflect both biological adaptation and model-specific constraints.

Ki-67-positive nuclei remained within the predefined low category in every primary tumor and retained 3D-OTC time point, although individual matched fragments varied. Conventional PA typically shows a low Ki-67 labeling index (< 5%)^28^, whereas higher indices are more frequently reported in recurrent PA and carcinoma ex pleomorphic adenoma^29–31^. The present data therefore support persistence of a low proliferative fraction within the sampled viable regions and provide no evidence of progressive enrichment for highly proliferative cells. However, Ki-67 is not a direct viability assay and cannot establish uniform survival throughout fragments approximately 3 mm in thickness. Focal central degenerative or necrotic-appearing areas were observed in some 3D-OTCs and excluded from scoring. Such a pattern is compatible with diffusion constraints in thicker ex vivo fragments^12^, but the non-viable fraction was not quantified and a global assessment of fragment viability is therefore not possible. Future optimization could focus on improved oxygen and nutrient delivery, for example through thinner fragments or perfusion/microfluidic support^32^.

PLAG1 remained detectable in all six cases at baseline and both retained culture time points. Cohort-level positivity remained high at day 14, followed by a lower mean and greater inter-case dispersion at day 21; the representative PA1 and PA4 series illustrate stable and variable fragment-level patterns, respectively. PLAG1 is a widely used diagnostic marker for PA and is linked to characteristic molecular alterations, including rearrangements that contribute to PA pathogenesis^33,34^. Multiple studies have highlighted PLAG1 IHC utility and its value in differentiating PA from other basaloid salivary neoplasms^16,35^. Persistence of PLAG1 positivity within the evaluated viable compartments supports retention of a tumor-associated nuclear phenotype, but the day-21 variability argues against describing expression as uniformly stable. From a practical perspective, PLAG1 may serve as one component of quality control in longitudinal 3D-OTC studies when interpreted together with morphology and other lineage-associated markers. Differences between independently sampled matched fragments must nevertheless be considered before attributing an individual change solely to culture conditions.

PanCK was strongly positive at baseline and remained detectable in most cases but showed moderate decline in some and loss in one case by day 21. E-cadherin membranous staining remained detectable but varied between cases; in some fragments, higher score categories were observed at later time points. These variable trajectories align with the established biphasic organization of PA, in which ductal epithelial structures coexist with myoepithelial-like components embedded in a variably myxoid/chondromyxoid stroma^36,37^. The relative proportion of ductal versus myoepithelial compartments can differ substantially within a tumor. Consequently, epithelial marker distribution (PanCK) and junctional marker intensity (E-cadherin) may vary spatially even in vivo. Ex vivo organotypic systems retain architecture, but the use of separate fragments at successive time points also makes fragment-to-fragment differences particularly relevant^12,38,39^. Each sampled piece may contain different proportions of epithelial and stromal components, which can influence the apparent marker profile. A plausible, but untested, explanation for reduced or lost PanCK in selected cases is that prolonged culture generates diffusion-limited oxygen and nutrient gradients. Densely cellular epithelial regions may be more affected, shifting the apparent balance between epithelial subcompartments within individual fragments^40,41^. Reduced cytokeratin staining could also reflect a change in differentiation state rather than outright loss of tumor cells, especially in a tumor with pronounced epithelial–myoepithelial plasticity. For E-cadherin, persistent membranous staining in most fragments suggests that epithelial cohesion was retained in at least part of the tumor cell population. Higher scores in selected later fragments could reflect adaptation in viable regions with reinforcement of junctional complexes, whereas weaker patterns could reflect reduced junctional organization or a lower ductal contribution^21,41–43^. Because successive time points represent different fragments of heterogeneous tumors, these mechanistic explanations remain hypotheses rather than demonstrated culture effects.

In our 3D-OTCs, α-SMA was absent or low in most cases and retained time points, while PA5 represented the strongest positive baseline pattern and PA6 remained negative. An α-SMA-positive vascular profile in the PA6 primary tumor served as an internal positive control, supporting the validity of the negative tumor-cell staining in that section. In contrast, p63 was detectable in five of six cases at baseline and both retained culture time points, although scores varied among matched fragments; PA6 remained negative. Myoepithelial-associated marker expression is known to be heterogeneous in PA, reflecting variability in both the contribution and differentiation state of the myoepithelial compartment across tumors and within individual lesions^23,44^. p63 is commonly used to highlight basal/myoepithelial differentiation in salivary gland tumors, including PA, and broad expression patterns have been documented^24,45^. In our model, p63 was more consistently detectable than α-SMA. This difference may be biologically meaningful, although the present IHC data cannot establish its mechanism. α-SMA primarily reflects mature myoepithelial differentiation and cytoplasmic contractile features, whereas p63 is a nuclear lineage marker associated with basal or progenitor-like epithelial programs and may therefore be less susceptible to culture-associated phenotypic drift^46,47^. Accordingly, p63 may be the more robust marker for tracking basaloid/myoepithelial-associated populations in ex vivo–cultured PA, whereas α-SMA requires cautious interpretation with respect to both biological heterogeneity and fragment composition.

PA is generally regarded as an immune-poor tumor, and the CD45 findings should be interpreted in that context. Low numbers of CD45-positive cells were detected in all sampled baseline fragments and remained detectable in the sampled viable compartments at both retained culture time points, with marked variation between cases and matched fragments. Emerging data indicate that PA can contain immune populations: single-cell profiling has identified myeloid cells, NK/T cells, and B cells in primary PA tissue^48^, and occasional PAs with prominent lymphoid infiltrates have also been described^49^. The exact central-versus-peripheral origin of our fragments was not documented, so the present data can neither confirm nor exclude preferential sampling near a tumor edge. Moreover, because the 3D-OTCs were non-vascularized, immune recruitment and replenishment were not possible. A higher CD45-positive percentage in a later fragment therefore does not demonstrate an increase in the absolute immune-cell number; it may reflect differences in fragment composition, sampling location, or relative loss of other cell populations that changes the denominator. Persistent CD45 staining represents resident leukocytes carried into culture with the original tissue rather than preservation of a functional or dynamic immune microenvironment. Studies of immune–tumor interactions would require separately validated immune add-back or perfused systems.

SSTR2 was positive in five of six primary tumors but was less frequently and generally more weakly detected in matched cultured fragments at days 14 and 21. SSTR2 immunoreactivity was therefore not reliably maintained during long-term 3D-OTC culture. Our group’s prior work demonstrated that SSTR2 expression in PA can be substantial and can correlate with^68^Ga-DOTATOC PET/CT findings, supporting its relevance as an imaging target^9,10^. Other reports have also described DOTATOC uptake in PA and highlight broader concepts of SSTR2-based imaging^11^. The differences between baseline and cultured fragments suggest that SSTR2 is phenotype-sensitive and may depend on microenvironmental cues that are altered ex vivo. Possible contributors include receptor downregulation, altered membrane trafficking under hypoxic or nutrient-limited conditions, and differences in the proportions of SSTR2-positive subcompartments between separate fragments. These mechanisms were not measured, and technical or sampling influences also remain possible. The data therefore do not establish a single culture-dependent mechanism. Rather than reducing the in vivo relevance of SSTR2, the findings define a boundary condition for receptor-target work in the present platform. Near-term optimization could focus on earlier assay windows, improved oxygen and nutrient delivery, and media conditions that better stabilize membrane receptor phenotypes. Functional receptor and penetration studies would be required before using the model for SSTR2-targeted imaging or therapeutic-response experiments.

A key next step is to define and improve transport and spatial viability within the cultured fragments. The present study did not directly measure oxygen, nutrient, or test-compound penetration, and future studies should combine tracer-based diffusion measurements with standardized whole-section mapping of viable and non-viable areas. Recent perfusion-based approaches for precision-cut tumor slices were developed to reduce diffusion gradients that arise in static air–liquid interface cultures^41,50^. Such systems may extend functional viability, while microfluidic platforms can enable spatially controlled perturbations on intact tissue slices^51^. A second priority is prospective allocation of separate patient-derived fragments for orthogonal RNA- and protein-based analyses, such as RT-PCR or immunoblotting, to distinguish biological changes from semi-quantitative IHC and sampling effects. Organotypic cultures can also be paired with molecular profiling before and after ex vivo exposure^50,52^. Future validation should incorporate salivary-gland-derived stromal and endothelial components, stem/progenitor-associated markers, and specimens from recurrent PA and carcinoma ex pleomorphic adenoma. These extensions require prospective tissue allocation and dedicated experiments but would substantially broaden the biological and translational scope of the platform.

### Limitations

This study has several limitations. The cohort was small (*n* = 6) and included only primary histologically benign PA; recurrent PA and carcinoma ex pleomorphic adenoma were not represented. The dermal fibroblast-based scaffold does not reproduce salivary-gland-specific stroma, and the exact central-versus-peripheral origin of individual tumor fragments was not documented. Separate physical fragments derived from the same patient were harvested at each time point, making fragment-to-fragment heterogeneity an inherent consideration. Scoring was restricted to three randomly selected high-power fields within morphologically viable, tumor-containing regions and excluded necrotic or degenerative areas; consequently, the results characterize surviving evaluable compartments rather than the complete fragment. The fraction of non-viable tissue was not prospectively quantified, and diffusion of oxygen, nutrients, or therapeutic compounds across the approximately 3-mm fragment thickness was not measured. The analyses relied on semi-quantitative IHC without digital whole-slide quantification, lineage co-staining for Ki-67, orthogonal RNA/protein validation, or stem/progenitor markers. Finally, the cultures were non-vascularized and cannot model immune recruitment or systemic influences. These limitations preclude claims of uniform long-term whole-fragment viability or validated therapeutic-response modeling.

## Conclusions

In this study, we established a patient-derived 3D-OTC platform that allows longitudinal assessment of PA histomorphology and selected biomarkers in surviving, morphologically viable tissue compartments for up to 21 days. Across baseline and matched fragments harvested at days 14 and 21, characteristic PA architecture, a low Ki-67-positive nuclear fraction, and PLAG1 positivity remained detectable, while epithelial, myoepithelial-associated, CD45, and SSTR2 readouts showed marker-, case-, and fragment-dependent patterns. The platform therefore provides a useful and defensible model for descriptive histomorphologic and biomarker-stability studies in viable compartments, while also revealing important limitations for phenotype-sensitive markers such as SSTR2 and for therapeutic-response applications. Direct diffusion and spatial viability measurements, salivary-specific stromal refinement, orthogonal assays, and inclusion of recurrent PA or carcinoma ex pleomorphic adenoma are required before broader translational use.

## Supplementary Information

Below is the link to the electronic supplementary material.

Supplementary Material 1

## Data Availability

All data generated or analyzed during this study are included in the published article and its supplementary information or will be available from the corresponding author upon reasonable request.
