# Supplementary material for: Patient-derived 3D organotypic co-cultures as a long-term ex vivo model for pleomorphic adenoma of the parotid gland
Source: Sci Rep. 2026 Sep 13;16:28499. doi: 10.1038/s41598-026-71512-3 (PMC13572365; doi:10.1038/s41598-026-71512-3)
Supplement: Supplementary file 1 [file 41598_2026_71512_MOESM1_ESM.docx]

**Supplementary Information**

**Table S1.** Demographic characteristics of included cases.

| **Case ID** | **Sex** | **Age at diagnosis (years)** |
| --- | --- | --- |
| PA1 | Male | 69 |
| PA2 | Female | 75 |
| PA3 | Female | 80 |
| PA4 | Female | 62 |
| PA5 | Male | 46 |
| PA6 | Male | 41 |

All cases were consecutive primary PAs of the parotid gland resected at initial surgery. Abbreviation: PA, pleomorphic adenoma.

**Table S2.** Primary antibodies used for IHC.

| **Antigen** | **Clone** | **Host species** | **Manufacturer** | **Catalog no.** | **Dilution** |
| --- | --- | --- | --- | --- | --- |
| Ki-67 | MIB-1 | Mouse | Dako, Denmark | M724001 | 1:50 |
| PLAG1 | Polyclonal | Rabbit | OriGene Technologies, USA | TA367737 | 1:30 |
| PanCK | MNF116 | Mouse | BioSystems S.A., Spain | Mob-052-05 | 1:200 |
| E-cadherin | 24E10 | Rabbit | Cell Signaling Technology, USA | 3195S | 1:200 |
| α-SMA | Polyclonal | Rabbit | Abcam, UK | ab5694 | 1:500 |
| p63 | SFI-6 | Mouse | DCS, Germany | PI006C002 | 1:100 |
| CD45 | D9M8I | Rabbit | Cell Signaling Technology, USA | 13917 | 1:200 |
| SSTR2 | UMB1 | Rabbit | Abcam, UK | ab134152 | 1:150 |

All antibodies were used for IHC on FFPE sections. Abbreviations: IHC, immunohistochemistry; PLAG1, pleomorphic adenoma gene 1; PanCK, pan-cytokeratin; α-SMA, alpha-smooth muscle actin; SSTR2, somatostatin receptor 2; FFPE, formalin-fixed paraffin-embedded.

**Table S3.** Individual immunohistochemical measurements and categorical scores across culture time points.

| **Case ID** | **Time point** | **Ki-67 (%)** | **PLAG1** | **PanCK** | **E-cadherin** | **α-SMA** | **p63** | **CD45** | **SSTR2** |
| --- | --- | --- | --- | --- | --- | --- | --- | --- | --- |
| PA1 | Primary tumor (day 0) | 0.0 | +++ (95.0%) | +++ (98.3%) | + | − (0.0%) | + | + (3.0%) | +++ |
| PA1 | OTC day 14 | 0.7 | +++ (93.3%) | +++ (97.0%) | +++ | − (0.0%) | + | ++ (19.7%) | + |
| PA1 | OTC day 21 | 0.0 | +++ (95.7%) | +++ (78.3%) | +++ | − (0.0%) | ++ | + (7.0%) | ++ |
| PA2 | Primary tumor (day 0) | 2.7 | +++ (88.3%) | +++ (99.0%) | + | − (0.0%) | + | + (4.3%) | +++ |
| PA2 | OTC day 14 | 0.0 | +++ (90.0%) | ++ (15.0%) | +++ | − (0.0%) | +++ | ++ (10.0%) | − |
| PA2 | OTC day 21 | 0.3 | ++ (80.0%) | +++ (92.0%) | +++ | + (1.3%) | + | + (9.3%) | + |
| PA3 | Primary tumor (day 0) | 1.7 | +++ (95.7%) | +++ (100.0%) | + | + (4.7%) | +++ | + (8.7%) | + |
| PA3 | OTC day 14 | 0.7 | +++ (95.7%) | +++ (96.7%) | ++ | − (0.0%) | +++ | + (1.3%) | − |
| PA3 | OTC day 21 | 1.0 | ++ (77.5%) | − (0.0%) | ++ | − (0.0%) | +++ | ++ (31.7%) | − |
| PA4 | Primary tumor (day 0) | 0.3 | +++ (95.0%) | +++ (98.3%) | + | + (1.7%) | + | + (5.3%) | ++ |
| PA4 | OTC day 14 | 1.0 | +++ (92.5%) | +++ (96.0%) | + | − (0.0%) | + | + (2.0%) | − |
| PA4 | OTC day 21 | 2.3 | + (47.5%) | ++ (26.7%) | +++ | − (0.0%) | + | ++ (21.0%) | + |
| PA5 | Primary tumor (day 0) | 0.7 | +++ (97.0%) | +++ (90.0%) | +++ | +++ (83.3%) | +++ | + (7.3%) | − |
| PA5 | OTC day 14 | 1.0 | +++ (96.3%) | +++ (75.0%) | ++ | ++ (18.3%) | + | + (2.7%) | − |
| PA5 | OTC day 21 | 3.0 | +++ (100.0%) | +++ (80.0%) | +++ | ++ (10.7%) | + | ++ (18.3%) | − |
| PA6 | Primary tumor (day 0) | 0.0 | ++ (75.0%) | +++ (66.7%) | + | − (0.0%) | − | + (1.7%) | + |
| PA6 | OTC day 14 | 0.0 | +++ (86.7%) | ++ (17.3%) | + | − (0.0%) | − | + (1.7%) | + |
| PA6 | OTC day 21 | 0.0 | ++ (55.0%) | ++ (33.3%) | + | − (0.0%) | − | + (1.0%) | − |

Values represent specimen-level means across three randomly selected high-power fields within morphologically viable, tumor-containing regions. Necrotic or degenerative areas were excluded from scoring. For PLAG1, PanCK, α-SMA, and CD45, cells show the categorical score followed by the corresponding mean percentage in parentheses. E-cadherin, p63, and SSTR2 are reported using their marker-specific ordinal scores because these scores incorporate staining intensity and/or membranous distribution and cannot be derived from percentages alone. Ki-67 is shown as the mean percentage of positive cell nuclei and categorized as low, intermediate, or high rather than −/+/++/+++. Score definitions: −, negative; +, weak/low; ++, moderate; +++, strong/high, according to the marker-specific criteria described in Methods.

**Table S4.** Descriptive summary of immunohistochemical measurements and categorical scores.

| **Marker** | **Time point** | **n** | **Mean % ± SD** | **Median % (range)** | **Mean score ± SD** | **Median score (range)** | **Detectable/positive n/6** | **Score distribution** |
| --- | --- | --- | --- | --- | --- | --- | --- | --- |
| Ki-67 | Primary tumor (day 0) | 6 | 0.89 ± 1.07 | 0.50 (0.00–2.67) | n.a. | n.a. | 4/6 | Low/Int./High = 6/0/0 |
| Ki-67 | OTC day 14 | 6 | 0.56 ± 0.46 | 0.67 (0.00–1.00) | n.a. | n.a. | 4/6 | Low/Int./High = 6/0/0 |
| Ki-67 | OTC day 21 | 6 | 1.11 ± 1.28 | 0.67 (0.00–3.00) | n.a. | n.a. | 4/6 | Low/Int./High = 6/0/0 |
| PLAG1 | Primary tumor (day 0) | 6 | 91.00 ± 8.40 | 95.00 (75.00–97.00) | 2.83 ± 0.41 | 3.0 (2–3) | 6/6 | −/+/++/+++ = 0/0/1/5 |
| PLAG1 | OTC day 14 | 6 | 92.42 ± 3.62 | 92.92 (86.67–96.33) | 3.00 ± 0.00 | 3.0 (3–3) | 6/6 | −/+/++/+++ = 0/0/0/6 |
| PLAG1 | OTC day 21 | 6 | 75.94 ± 21.14 | 78.75 (47.50–100.00) | 2.17 ± 0.75 | 2.0 (1–3) | 6/6 | −/+/++/+++ = 0/1/3/2 |
| PanCK | Primary tumor (day 0) | 6 | 92.06 ± 12.95 | 98.33 (66.67–100.00) | 3.00 ± 0.00 | 3.0 (3–3) | 6/6 | −/+/++/+++ = 0/0/0/6 |
| PanCK | OTC day 14 | 6 | 66.17 ± 39.63 | 85.50 (15.00–97.00) | 2.67 ± 0.52 | 3.0 (2–3) | 6/6 | −/+/++/+++ = 0/0/2/4 |
| PanCK | OTC day 21 | 6 | 51.72 ± 36.80 | 55.83 (0.00–92.00) | 2.17 ± 1.17 | 2.5 (0–3) | 5/6 | −/+/++/+++ = 1/0/2/3 |
| E-cadherin | Primary tumor (day 0) | 6 | n.a. | n.a. | 1.33 ± 0.82 | 1.0 (1–3) | 6/6 | −/+/++/+++ = 0/5/0/1 |
| E-cadherin | OTC day 14 | 6 | n.a. | n.a. | 2.00 ± 0.89 | 2.0 (1–3) | 6/6 | −/+/++/+++ = 0/2/2/2 |
| E-cadherin | OTC day 21 | 6 | n.a. | n.a. | 2.50 ± 0.84 | 3.0 (1–3) | 6/6 | −/+/++/+++ = 0/1/1/4 |
| α-SMA | Primary tumor (day 0) | 6 | 14.94 ± 33.55 | 0.83 (0.00–83.33) | 0.83 ± 1.17 | 0.5 (0–3) | 3/6 | −/+/++/+++ = 3/2/0/1 |
| α-SMA | OTC day 14 | 6 | 3.06 ± 7.48 | 0.00 (0.00–18.33) | 0.33 ± 0.82 | 0.0 (0–2) | 1/6 | −/+/++/+++ = 5/0/1/0 |
| α-SMA | OTC day 21 | 6 | 2.00 ± 4.28 | 0.00 (0.00–10.67) | 0.50 ± 0.84 | 0.0 (0–2) | 2/6 | −/+/++/+++ = 4/1/1/0 |
| p63 | Primary tumor (day 0) | 6 | n.a. | n.a. | 1.50 ± 1.22 | 1.0 (0–3) | 5/6 | −/+/++/+++ = 1/3/0/2 |
| p63 | OTC day 14 | 6 | n.a. | n.a. | 1.50 ± 1.22 | 1.0 (0–3) | 5/6 | −/+/++/+++ = 1/3/0/2 |
| p63 | OTC day 21 | 6 | n.a. | n.a. | 1.33 ± 1.03 | 1.0 (0–3) | 5/6 | −/+/++/+++ = 1/3/1/1 |
| CD45 | Primary tumor (day 0) | 6 | 5.06 ± 2.63 | 4.83 (1.67–8.67) | 1.00 ± 0.00 | 1.0 (1–1) | 6/6 | −/+/++/+++ = 0/6/0/0 |
| CD45 | OTC day 14 | 6 | 6.22 ± 7.35 | 2.33 (1.33–19.67) | 1.33 ± 0.52 | 1.0 (1–2) | 6/6 | −/+/++/+++ = 0/4/2/0 |
| CD45 | OTC day 21 | 6 | 14.72 ± 11.10 | 13.83 (1.00–31.67) | 1.50 ± 0.55 | 1.5 (1–2) | 6/6 | −/+/++/+++ = 0/3/3/0 |
| SSTR2 | Primary tumor (day 0) | 6 | n.a. | n.a. | 1.67 ± 1.21 | 1.5 (0–3) | 5/6 | −/+/++/+++ = 1/2/1/2 |
| SSTR2 | OTC day 14 | 6 | n.a. | n.a. | 0.33 ± 0.52 | 0.0 (0–1) | 2/6 | −/+/++/+++ = 4/2/0/0 |
| SSTR2 | OTC day 21 | 6 | n.a. | n.a. | 0.67 ± 0.82 | 0.5 (0–2) | 3/6 | −/+/++/+++ = 3/2/1/0 |

Statistics were calculated across the six independent biological cases using specimen-level mean values. No inferential hypothesis testing was performed. Baseline, day 14, and day 21 constitute the primary longitudinal comparison. For markers with applicable ordinal scores, categories were numerically encoded for secondary descriptive purposes only (0 = −, 1 = +, 2 = ++, 3 = +++); because these scores are ordinal, medians, ranges, and category distributions should be prioritized over score means. Ki-67 is summarized as a percentage with low/intermediate/high categories; “n.a.” is shown where a −/+/++/+++ ordinal score does not apply. For E-cadherin, p63, and SSTR2, percentage summaries are reported as “n.a.” because the corresponding individual percentages are not provided in Table S3 and the final scores incorporate intensity and/or staining distribution. For Ki-67, “detectable” denotes a mean value >0%; for markers with ordinal scores, “positive” denotes a score >0.

**Supplementary Figure S1**

**Supplementary Figure S1. Additional complete immunohistochemical series illustrating inter-case variability.** **(A)** PanCK in PA3, **(B)** E-cadherin in PA6, and **(C)** p63 in PA6 at baseline (primary tumor, day 0) and OTC days 14 and 21. Scale bars, 60 µm.
